# Analysis of linear electrode array EMG for assessment of hemiparetic biceps brachii muscles

**DOI:** 10.3389/fnhum.2015.00569

**Published:** 2015-10-23

**Authors:** Bo Yao, Xu Zhang, Sheng Li, Xiaoyan Li, Xiang Chen, Cliff S. Klein, Ping Zhou

**Affiliations:** ^1^Biomedical Engineering Program, University of Science and Technology of ChinaHefei, China; ^2^Department of Physical Medicine and Rehabilitation, University of Texas Health Science Center, and TIRR Memorial Hermann Research Center, HoustonTX, USA; ^3^Guangdong Work Injury Rehabilitation CenterGuangzhou, China

**Keywords:** EMG, linear electrode array, stroke, power spectrum, isometric contraction

## Abstract

This study presents a frequency analysis of surface electromyogram (EMG) signals acquired by a linear electrode array from the biceps brachii muscles bilaterally in 14 hemiparetic stroke subjects. For different levels of isometric contraction ranging from 10 to 80% of the maximum voluntary contraction (MVC), the power spectra of 19 bipolar surface EMG channels arranged proximally to distally along the muscle fibers were examined in both paretic and contralateral muscles. It was found that across all stroke subjects, the median frequency (MF) and the mean power frequency (MPF), averaged from different surface EMG channels, were significantly smaller in the paretic muscle compared to the contralateral muscle at each of the matched percent MVC contractions. The muscle fiber conduction velocity (MFCV) was significantly slower in the paretic muscle than in the contralateral muscle. No significant correlation between the averaged MF, MPF, or MFCV vs. torque was found in both paretic and contralateral muscles. However, there was a significant positive correlation between the global MFCV and MF. Examination of individual EMG channels showed that electrodes closest to the estimated muscle innervation zones produced surface EMG signals with significantly higher MF and MPF than more proximal or distal locations in both paretic and contralateral sides. These findings suggest complex central and peripheral neuromuscular alterations (such as selective loss of large motor units, disordered control of motor units, increased motor unit synchronization, and atrophy of muscle fibers, etc.) which can collectively influence the surface EMG signals. The frequency difference with regard to the innervation zone also confirms the relevance of electrode position in surface EMG analysis.

## Introduction

Stroke is a common neurologic disorder in adults which can lead to severe disability. Approximately 700,000 strokes occur annually in the United States (Broderick et al., 1998), and about 15 million people worldwide suffer from stroke each year (Mackay and Mensah, 2004). After a stroke, motor impairments such as spasticity, weakness, and abnormal movement coordination of the upper and lower limbs contralateral to the brain lesion usually occur (Andrews and Bohannon, 2000). In order to explore neuromuscular changes in stroke survivors, electromyogram (EMG) techniques have been used, which provide valuable information about deterioration of muscle function including changes in motor unit and muscle fiber properties.

Electromyogram power spectral analysis is often used for assessment of neuromuscular function in both healthy and diseased states. Spectral alternations have been found to be associated with changes in both peripheral (e.g., loss of muscle fibers and functioning motor units, changes of motor unit type composition, etc.) and central (e.g., disordered control of motor units, increased synchronization, etc.) factors. To examine neuromuscular changes following a stroke, the EMG power spectrum from paretic and contralateral muscles has been compared, and the findings are inconclusive. A previous study reported that there was a power density increase in the relatively low spectral frequency component in paretic biceps brachii muscle (compared with the contralateral muscle) of one stroke subject, whereas no significant spectrum change was observed in the other five tested subjects (Gemperline et al., 1995). Others studied power spectral analysis of surface motor unit action potentials (MUAPs) from the biceps brachii muscle of stroke subjects (Kallenberg and Hermens, 2009). They found that the mean power frequency (MPF) of the MUAP spectrum was smaller in the paretic muscle compared with the contralateral muscle. However, no significant MPF difference was found between the paretic and contralateral muscles in the same group of subjects, based on an analysis of the surface EMG interference pattern (Kallenberg and Hermens, 2009). In a recent study of the first dorsal interosseous (FDI) muscle post-stroke, it was found that there was a significant reduction in MPF in the paretic muscle compared with the contralateral muscle (Li et al., 2013).

In addition to mixed observations on EMG spectral alterations post-stroke, the relationship between the surface EMG spectrum and contraction force also appeared to be complex. Few have studied the relationship between the surface EMG spectrum and force in stroke subjects. A previous study reported that the surface EMG median frequency (MF) slightly decreased in paretic and contralateral biceps brachii with increased contraction force in stroke subjects, whereas no statistical significance was found between MF and force (Hu et al., 2007). In a recent study, we also found no correlation between MPF and contraction force from the FDI muscle in either paretic or contralateral hand in stroke subjects (Li et al., 2013). The relationship between EMG spectrum and force is also uncertain in healthy control subjects. For example, two studies reported an increase in biceps brachii EMG center frequency (MPF or MF) with increased isometric torque (Moritani and Muro, 1987; Kaplanis et al., 2009). However, Komi and Vitasalo (1976) reported that the rectus femoris EMG center frequency decreased with increasing muscle force. In contrast, Coburn et al. (2005) found no significant correlation between vastus medialis EMG MPF and torque, and Beck et al. (2008) also reported that there was no consistent pattern for the MPF vs. isometric torque relationship.

Most of the previous studies that employed surface EMG power spectral analysis were performed with single channel conventional surface electrodes. In the past decade, advances in high density surface EMG using electrode arrays have been made, which allow recording over a large portion of a muscle (Drost et al., 2006; Merletti et al., 2008). Among different electrode array designs, a 1-dimensional linear electrode array is relatively simple and convenient to use. It can simultaneously record EMG from different locations along the muscle surface and has many useful applications including the identification of innervation zones and estimation of muscle fiber conduction velocity (MFCV), and is particularly suitable for single pennate biceps brachii muscle (Merletti et al., 2003).

The current study presents a comprehensive analysis of linear electrode array EMG signals of the biceps brachii muscles in stroke subjects. We examined whether a significant difference in EMG power spectrum can be observed between paretic and contralateral muscles, and the correlation between muscle contraction level and EMG power spectrum was explored. Taking advantage of a linear electrode array, we further examined whether there was a significant difference in MFCV between paretic and contralateral muscles. In addition, the EMG power spectrum from different electrode channels with respect to estimated location of the muscle innervation zone was also compared.

## Materials and Methods

### Subjects

Fourteen hemiparetic stroke survivors (5 female and 9 male, 41–89 years old) volunteered to participate in this study (**Table 1**). Inclusion criteria included: (i) an interval of at least 6 months post-stroke; (ii) hemiplegia secondary to an ischemic or hemorrhage stroke; (iii) Modified Ashworth Scale (MAS) score less than 3 in the elbow flexors on the paretic side; (iv) ability to follow commands and to follow a visual target; and (v) ability to sign an informed consent. The study was approved by Committee for the Protection of Human Subjects at the University of Texas Health Science Center at Houston and TIRR Memorial Hermann Hospital (Houston, USA). All procedures conformed to the Declaration of Helsinki, and all subjects signed the informed consent prior to any experiment procedures.

**Table 1:** Subject information.

Subject ID	Gender	Age	Paretic side	Modified Ashworth Scale (MAS)
1	Male	77	Right	1
2	Female	66	Right	1
3	Female	59	Left	1
4	Male	60	Left	0
5	Female	61	Left	2
6	Male	48	Left	0
7	Male	75	Left	1
8	Male	89	Right	1+
9	Male	51	Right	1
10	Male	67	Right	2
11	Male	78	Right	1+
12	Male	41	Right	1+
13	Female	67	Left	1
14	Female	51	Right	2

### Experiment Protocols

Subjects were seated comfortably in a height-adjustable chair. Their forearm and wrist were positioned on a customized apparatus, where the wrist was fixed using four vertical plates. The elbow joint was approximately 90°, with the shoulder slightly abducted (45°), and flexed (30°). At first the subjects were allowed to practice approximately 3–5 trials to be familiar with the experimental setting and tasks. The maximum voluntary contraction (MVC) was recorded in both paretic and contralateral muscles separately. Each subject performed two MVC trials of the biceps brachii muscle. The larger of the two MVC trials was defined as the MVC value. The final MVC value was marked as a horizontal line on the computer screen and subdivided to indicate the contraction levels of 10, 20, 30, 40, 50, 60, 70, and 80% of the MVC. During each trial, the subject was encouraged to reach the desired force target displayed on the monitor and maintain it as stable as possible during a 10-s trial, which started after the first computer-generated auditory tone (at 2 s) and stopped after a second tone (at 8 s). The trial sequence for different contraction levels was randomized. Each contraction level was repeated twice for both the paretic and contralateral sides. To avoid muscle fatigue, subjects were allowed sufficient rest between trials.

### EMG Acquisition

Surface EMG signals were recorded from the biceps brachii muscle using a customized linear electrode array consisting of 20 bar electrodes (1 mm width × 10mm length and 5 mm inter-electrode distance). The electrode array was secured with medical tape and special attention was paid to ensure the position was similar for the paretic and contralateral muscles. A reference electrode was placed on the lateral condyle of the tested arm. Surface EMG signals were recorded via the Porti EMG acquisition system (TMS International, The Netherlands). The sampling frequency was 2 kHz per channel and the bandwidth filter was set at 10–500 Hz. Torque signals were recorded by a sensor (Model TRS 500, Transducers Techniques, Temecula, CA, USA) at a sampling frequency of 1 kHz and digitized using a BNC-2090A data acquisition board (National Instruments, Austin, TX, USA). Both the EMG and torque data were stored on the computer for oﬄine analysis using a customized MATLAB program (MathWorks, Natick, MA, USA).

### Data Analysis

Prior to the analysis, 19-channel bipolar EMG signals were generated by subtracting each pair of the monopolar EMG signals from adjacent electrodes along the muscle fibers. Potential power line interference in surface EMG data was eliminated by a spectrum interpolation algorithm (Mewett et al., 2004).

**FIGURE 1:**
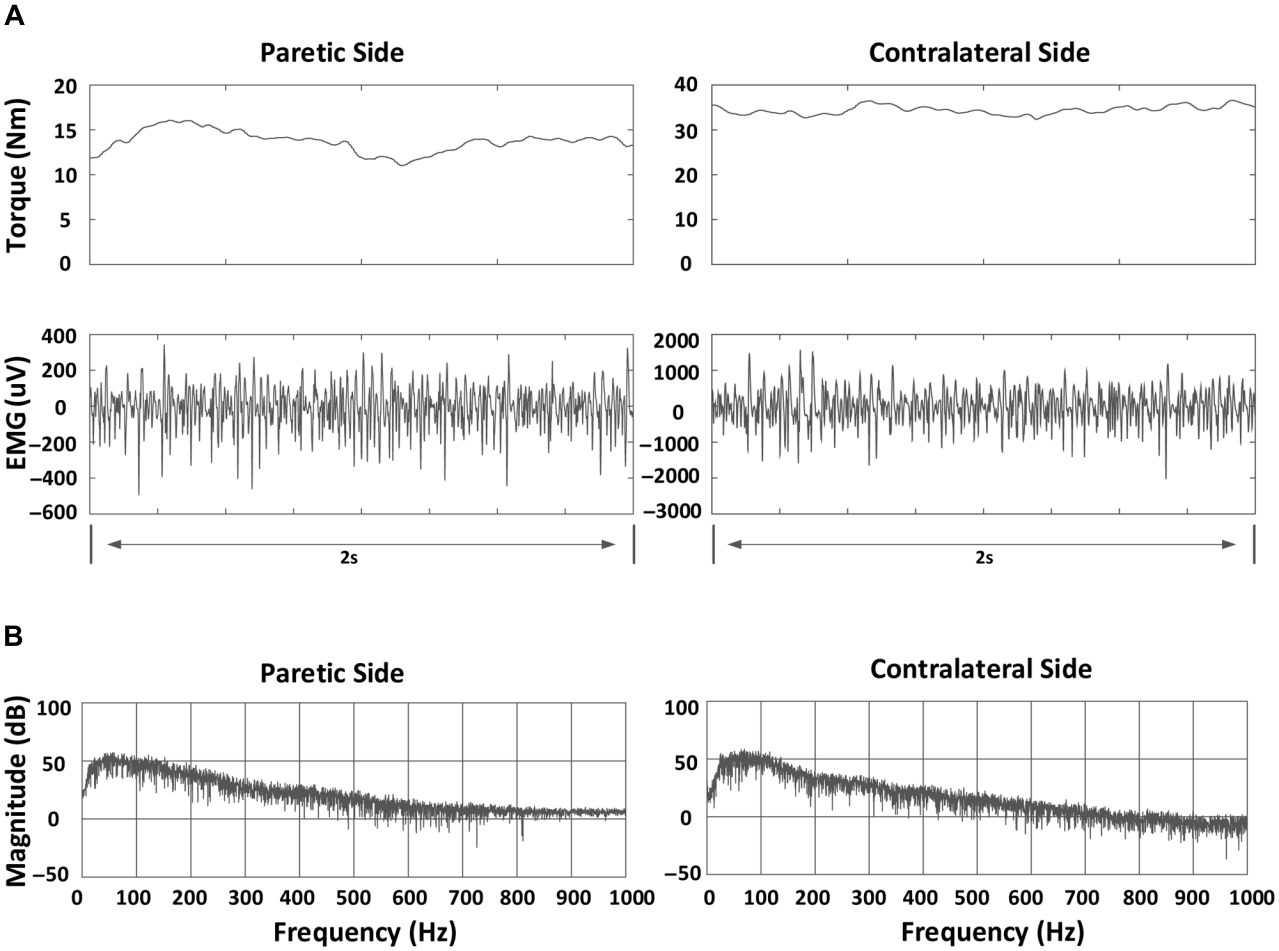
**An example of torque, bipolar electromyogram (EMG) signals and power spectrum for a stroke subject at 70% MVC in a channel (index: 10): **(A)** Torque and bipolar EMG signals in both paretic and contralateral sides.** A 2-s segment indicating relatively stable torque is shown; **(B)** Power spectrum of EMG signals in both paretic and contralateral sides.

Subsequently, MF, MPF, and MFCV were calculated for each EMG channel. A 2-s segment with relatively stable torque was selected (by observation of torque signals) at each contraction level (**Figure 1**). Then, for each selected data segment, power spectrum analysis was performed by applying fast Fourier transform (FFT) to individual surface EMG channels. The MPF and MF of the spectrum were calculated for each channel, respectively. The MFCV was estimated based on two single differential channels in the longitudinal direction of the biceps brachii muscle. MFCV was calculated as *d*/τ, where τ was the time delay between two signals detected *d* meters apart (Farina and Merletti, 2000). The two channels over or closest to the innervation zone were excluded for MFCV calculation. For bipolar EMG channels distal and proximal to the innervation zone, MFCV was calculated for each channel pair.

Once MF, MPF, and MFCV values were obtained from each channel (or channel pair), they were averaged for each contraction level. The relationship between each of the parameters (MF, MPF, and MFCV) and contraction level was determined. To simplify the comparison between paretic and contralateral muscles, the mean values of MF, MPF, or MFCV at each force level were further averaged to give global averages of the respective measures. To give further insight into the surface EMG frequency change, the grand-average power spectra of the surface EMG in both paretic and contralateral muscles were computed and compared. The power spectrum of surface EMG for each channel and torque level was normalized and averaged to form the grand-average power spectrum in both paretic and contralateral muscles.

**FIGURE 2:**
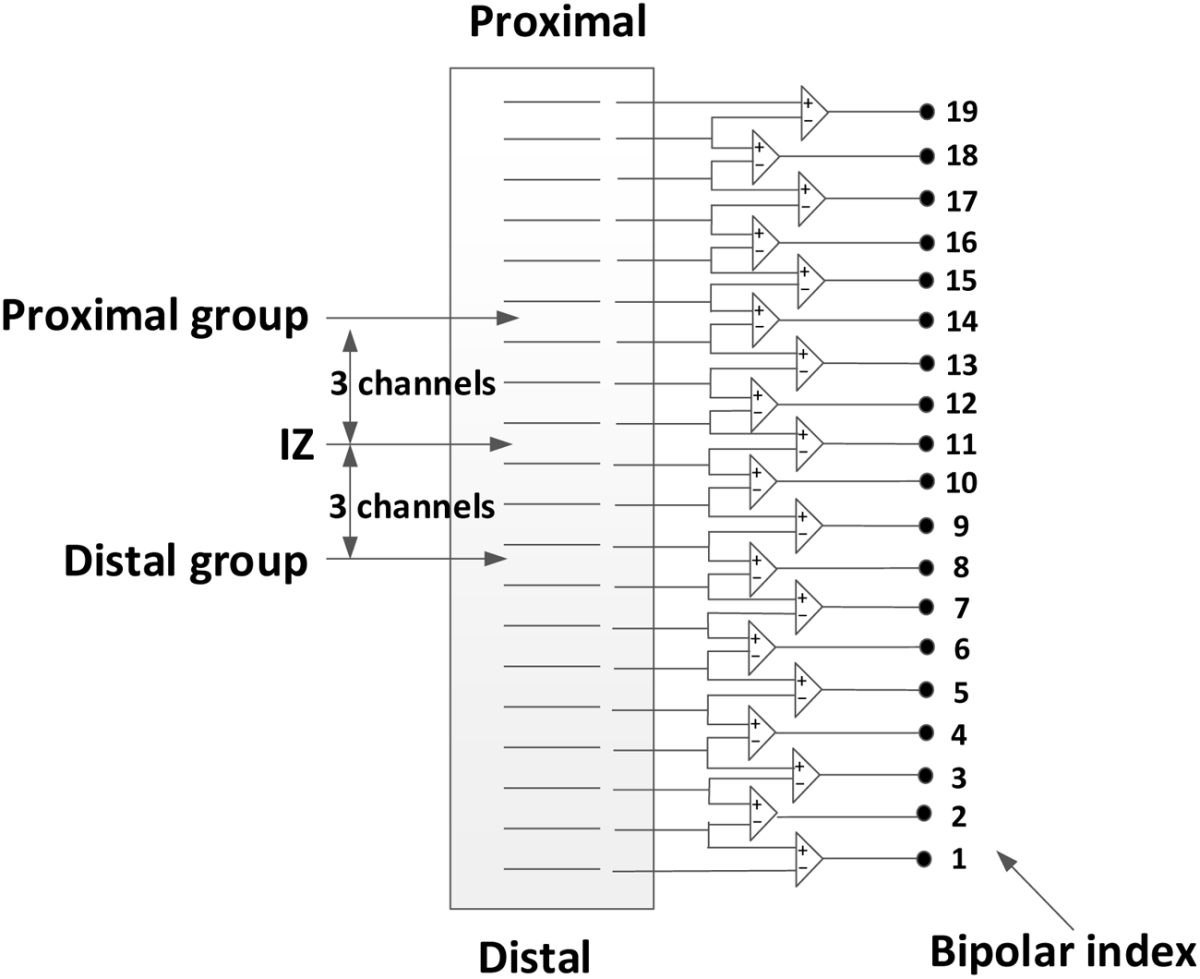
**Selection of three groups of surface EMG channels from the 20-channel linear electrode array: the over innervation zone group, the distal group, and the proximal group**.

The effect of electrode location (with respect to estimated muscle innervation zones) on the surface EMG power spectrum was also determined. First, the channel with minimum amplitude and phase reversal (on two sides of the channel) was selected as the estimated location of the innervation zone (Merletti et al., 1999; Beck et al., 2008). Then, three groups of surface EMG (each containing two bipolar EMG signals from three electrodes) were selected from the linear array for power spectrum comparison, as shown in **Figure 2**. The first group included two consecutive bipolar signals that were closest or directly over the innervation zone (yielding the minimum amplitude and phase reversal). The second group contained two consecutive bipolar signals, shifted 15 mm (equivalent to three channels and 5 mm interval between two consecutive channels) proximal to the estimated innervation zone and was referred to as the proximal group. Similarly, the third group contained two consecutive bipolar signals, shifted 15 mm distal to the estimated innervation zone and was referred to as the distal group. For each group, the MF and MPF of the surface EMG power spectrum were averaged across two bipolar signals as well as different force levels to obtain a representative value. The effect of the electrode position on surface EMG power spectrum was analyzed by comparing the representative MF and MPF values of the different groups.

### Statistical Analyses

A repeated measure two-way [side (paretic and contralateral) × torque (10, 20, 30, 40, 50, 60, 70, and 80% MVC)] analysis of variance (ANOVA) was used to analyze the MF, MPF, and MFCV data. Correlation between spectral parameters (MPF or MF) and the MFCV were examined. The statistical significance for the MF or MPF in different electrode locations (innervation zones and distal and proximal groups) was analyzed using two separate one-way repeated-measure ANOVA for both paretic and contralateral muscles (SPSS Inc., Chicago, IL, USA).

## Results

**FIGURE 3:**
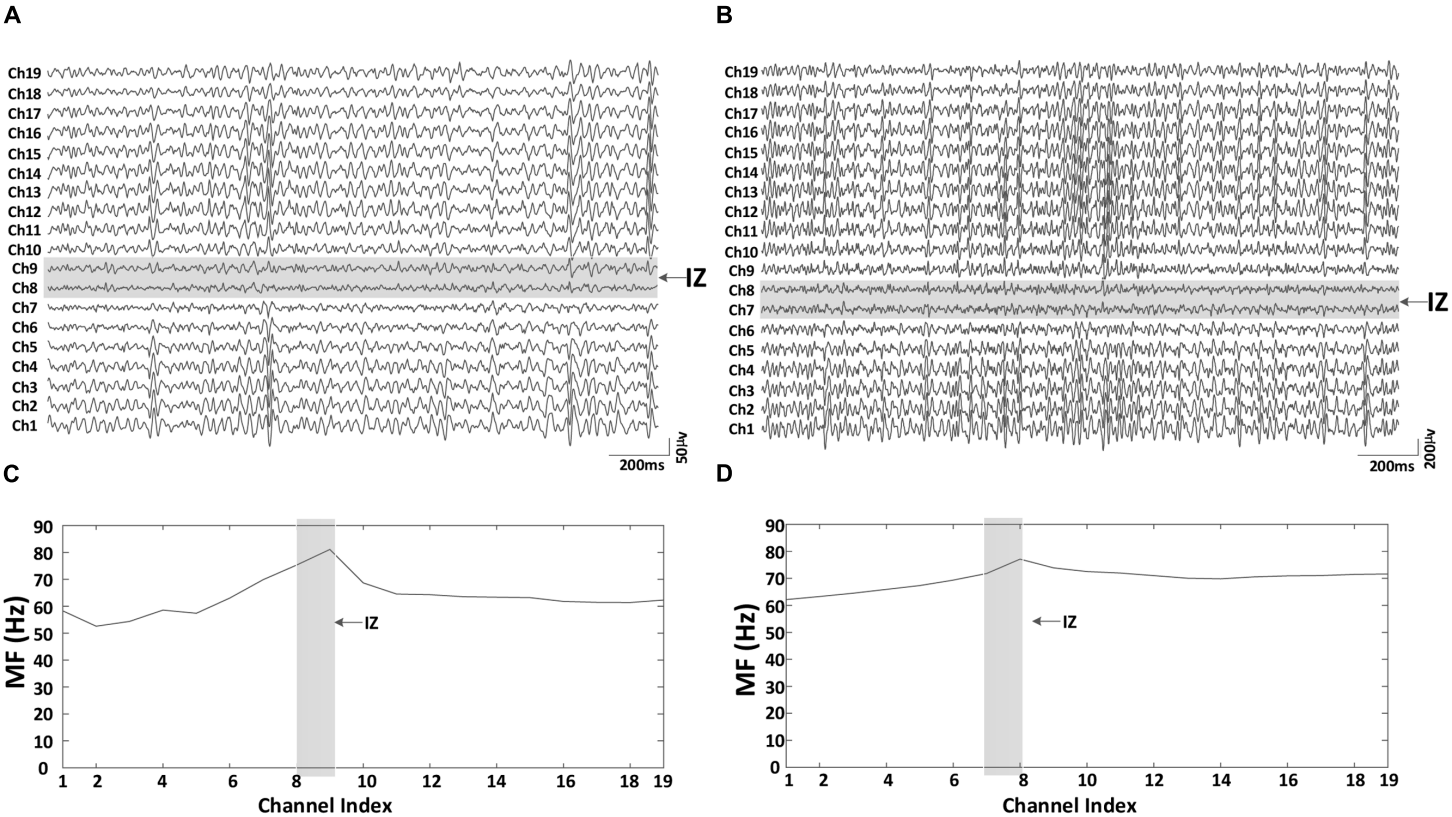
**An example of the 19 bipolar surface EMG signals and the corresponding MF for both paretic and contralateral muscles (Subject 1, 30% MVC). (A)** Paretic side EMG, **(B)** contralateral side EMG, **(C)** paretic side surface EMG median frequency (MF), and **(D)** contralateral side surface EMG MF. The two consecutive channels marked gray indicate the innervation zone. MF was largest for channels close to estimated innervation zone in the 19 bipolar channels for both paretic and contralateral muscles.

As expected, the paretic side (averaged MVC: 18.84 ± 12.22 Nm, mean ± SD) was significantly weaker than the contralateral side (averaged MVC: 36.92 ± 17.34 Nm; *F* = 22.10, *p* < 0.001). The coefficient of variation (CoV) of the torque on the paretic side (0.067 ± 0.016) was significantly higher than that on the contralateral side (0.044 ± 0.004; *F* = 28.47, *p* < 0.05). At the same level of muscle contraction (30% MVC), **Figure 3** shows an example of the surface EMG signals from different bipolar channels of the linear electrode array for both paretic and contralateral muscles (Subject 1) as well as their MF in each channel. Muscle innervation zones were estimated to be the channels demonstrating the minimum amplitude and phase reversal of the surface EMG traveling toward proximal and distal tendons (marked in gray in the figure). Correspondingly, **Figures 3C,D** indicate that there was a MF peak value for the channels closest to the estimated innervation zones. The surface EMG MF was largest for channels close to estimated innervation zones in the 19 bipolar channels for both paretic and contralateral muscles (paretic: 81.11 Hz, channel index: 9 and contralateral: 77.11 Hz, channel index: 8). Although the peak MF value in paretic side was larger than the contralateral side for this subject, the MF averaged from all the channels was smaller in the paretic side (63.42 ± 6.76 Hz) compared with the contralateral side (69.80 ± 3.71 Hz).

**FIGURE 4:**
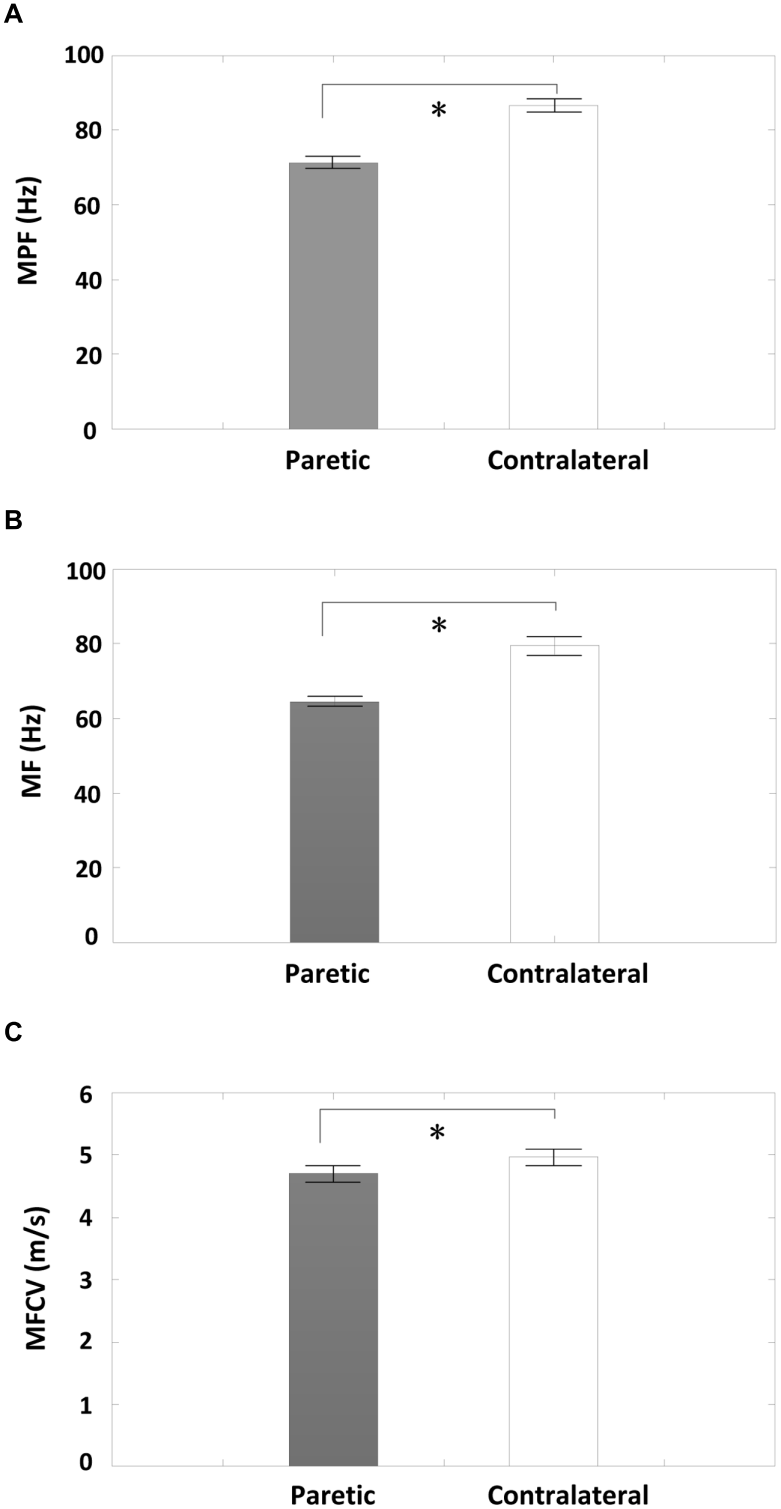
**A comparison of global averages of surface EMG MF, mean power frequency (MPF), and Muscle fiber conduction velocity (MFCV) measurements between the paretic and contralateral sides from all subjects. (A)** MPF, **(B)** MF, and **(C)** MFCV; ^∗^*p* < 0.05.

**FIGURE 5:**
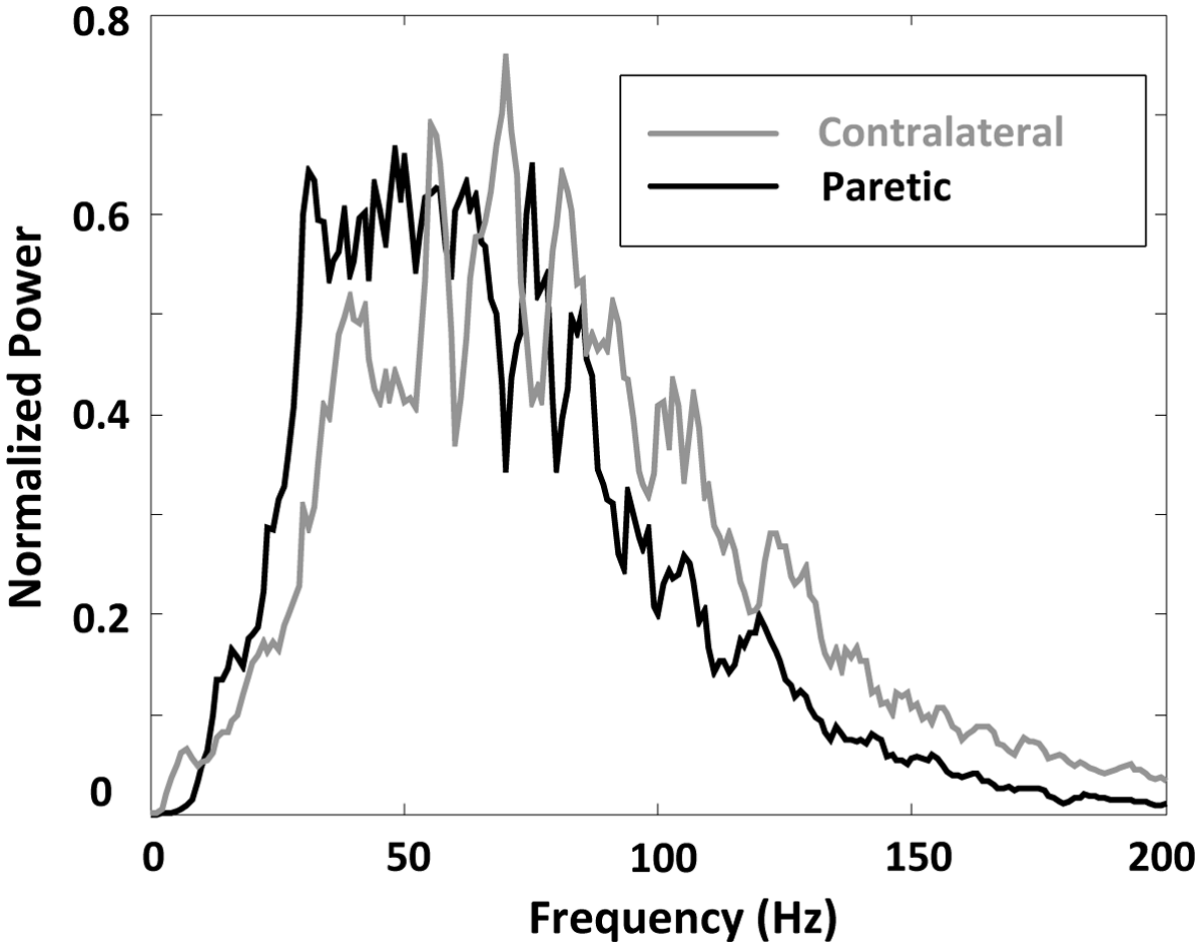
**A comparison of surface EMG grand-average power spectrum for paretic and contralateral muscles**.

**FIGURE 6:**
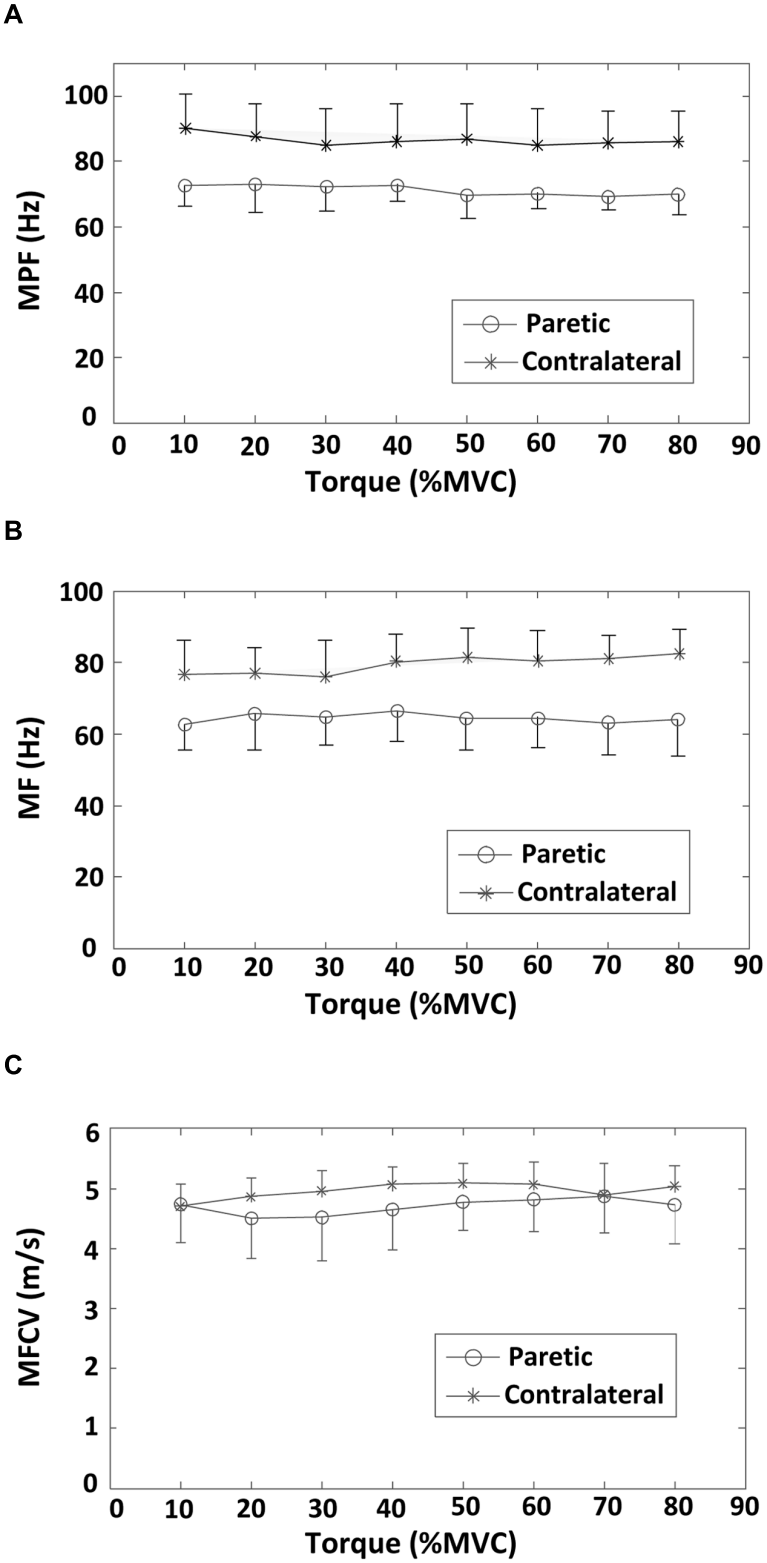
**Averaged surface EMG MF, MPF, and MFCV values across 19 bipolar EMG channels at different torque levels (from 10 to 80% MVC) for both paretic and contralateral sides. (A)** MPF, **(B)** MF, and **(C)** MFCV.

A comparison of surface EMG parameters between paretic and contralateral muscles is shown in **Figure 4**. The global MF value (paretic: 64.56 ± 1.32 Hz and contralateral: 79.46 ± 2.45 Hz; *F* = 28.51, *p* < 0.001), MPF value (paretic: 71.36 ± 1.58 Hz and contralateral: 86.60 ± 1.67 Hz; *F* = 115.42, *p* < 0.001), and MFCV value (paretic: 4.69 ± 0.13 m/s and contralateral: 4.96 ± 0.13 m/s; *F* = 15.15, *p* < 0.001) on the paretic side were significantly smaller than those on the contralateral side. **Figure 5** shows a grand-average power spectrum comparison between paretic and contralateral muscles from all the signals. **Figure 6** presents a more detailed comparison of the MF, MPF, and MFCV (averaged from different channels) at each specific muscle contraction level. The results indicate smaller MF, MPF, and MFCV (averaged from all the subjects) in the paretic side compared with the contralateral side across all force levels. For both the paretic and contralateral muscles, we did not observe a significant relationship between the MF, MPF, or MFCV and contraction level. There were significant positive correlations between the global MFCV vs. MF (*r* = 0.15, *p* < 0.05) and the global MPF vs. MF (*r* = 0.625, *p* < 0.001). No significant correlation was found between the global MFCV and MPF (*r* = 0.01, *p* > 0.05).

**FIGURE 7:**
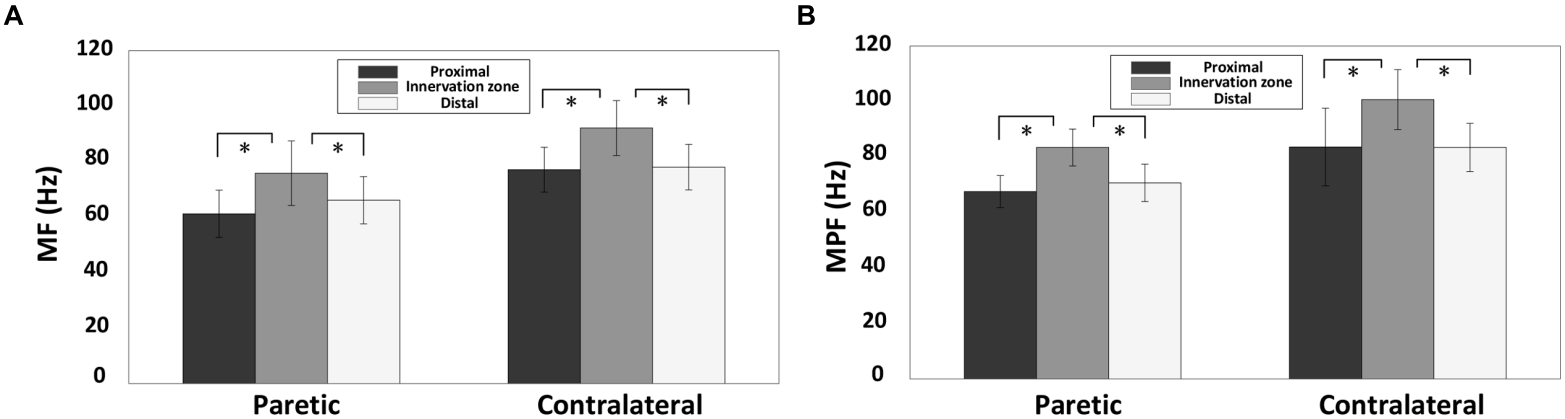
**A comparsion of surface EMG MF and MPF for the three electrode channel groups for both paretic and contralateral sides: the innervation zone group, the proximal group and the distal group.** The MF and MPF were averaged across different torques from all subjects. **(A)** MF and **(B)** MPF; ^∗^*p* < 0.05.

**Figure 7** shows the surface EMG MF and MPF comparison for the three channel groups (innervation zone, proximal, and distal groups), each containing two bipolar channels. The findings indicate a significantly larger MF for channels closest to the estimated innervation zones compared with more proximal or distal channels, for both paretic and contralateral muscles (paretic side, proximal: 61.18 ± 8.52 Hz, innervation zone: 75.86 ± 11.63 Hz, and distal: 66.09 ± 8.51 Hz; *F* = 8.365, *p* < 0.05; contralateral side, proximal: 77.10 ± 8.07 Hz, innervation zone: 92.12 ± 9.90 Hz, and distal: 77.99 ± 8.19 Hz; *F* = 34, *p* < 0.001; **Figure 7A**). The MPF results were consistent with MF (paretic side, proximal: 67.53 ± 5.78 Hz, innervation zone: 83.44 ± 6.65 Hz, and distal: 70.68 ± 6.73 Hz; *F* = 23.10, *p* < 0.001; contralateral side, proximal: 83.63 ± 14.3 Hz, innervation zone: 100.69 ± 10.8 Hz, and distal: 83.45 ± 8.67 Hz; *F* = 32.52, *p* < 0.001; **Figure 7B**). Consistent with the global differences in MPF and MF between sides (**Figures 5** and **6**), the MPF and MF were also smaller in the paretic compared to the contralateral side for the three channels groups.

## Discussion

The main findings of the present study include: (i) The EMG MF or MPF of the paretic biceps brachii muscle of stroke subjects was significantly smaller than that of the contralateral muscle. The MFCV was significantly slower in the paretic muscle compared with the contralateral muscle; (ii) No significant correlation was found between the MF, MPF, or MFCV vs. torque for both paretic and contralateral muscles; (iii) There was a significant difference in MF and MPF at the different electrode locations, with the channels over the innervation zone demonstrating significantly higher MF and MPF values than the distal and proximal channels for both paretic and contralateral muscles. (iv) There was a significant positive correlation between the global MFCV and MF.

The findings of this study indicate that the surface EMG power spectrum is likely affected by complex neuromuscular alterations post-stroke. The reduction in EMG MF or MPF in the paretic side compared to the contralateral side was also reported in several previous studies (Muro et al., 1982; Gemperline et al., 1995). Our recent investigation on the FDI muscle also reported decreased surface EMG MPF on the paretic compared with the contralateral side (Li et al., 2013). The reduction of the power spectrum observed on the paretic side might be due to various factors. For example, decreased EMG center frequency might be a result of selective degeneration of the larger motor units (Lukacs et al., 2008). In particular, the loss of larger motor units in more superficial regions of the muscle would be expected to significantly contribute to reduced EMG center frequencies. Alternatively, the altered EMG power spectrum might reflect muscle fiber atrophy. Atrophy may also reduce the MFCV of the paretic muscle (Blijham et al., 2006). Several studies have reported a linear relationship between MFCV and frequency of surface EMG in healthy subjects (Sadoyama et al., 1983; Arendt-Nielsen and Mills, 1985). We found significant positive correlations between the global MFCV and MF. The study of Toffola et al. (2001) reported a tendency toward type II fiber atrophy in paretic muscles of stroke survivors. Preferential type II fiber atrophy may result in surface EMG with the higher MF on the unaffected side than the paretic side. Increased fatty tissue infiltration with motor unit loss or muscle fiber atrophy may further reduce the high frequency components of paretic muscle. A reduction in motor unit firing rates (Gemperline et al., 1995; Li et al., 2015) might be another factor that contributes to a reduction in surface EMG center frequencies on paretic side. Reduced motor unit firing rates post-stroke were reported in several previous studies, possibly reflecting a decrease in descending excitation or synaptic input to the motoneuron pool (McComas et al., 1971; Murase et al., 2004). In addition, increased motor unit synchronization after stroke may further reduce the EMG power spectrum. However, it should be noted that after stroke, there are also factors which might increase the EMG center frequency, such as muscle fiber reinnervation demonstrated as increased muscle fiber density, neuromuscular jitter, and more complex MUAP waveforms (Lukacs et al., 2009). Thus, the observed MF, MPF, and MFCV reduction of the paretic muscle can be viewed as a collective effect of these factors. By solely using global surface EMG parameters, it is difficult to differentiate various factors contributing to surface EMG spectrum changes. This remains a limitation of the current study. Other techniques such as single motor unit analysis, high resolution imaging (for examination of muscle fiber changes) are required to examine the complex neuromuscular changes after stroke. In addition, the MFCV values reported in the study were relatively high, possibly due to limited time resolution (under 2 kHz sampling rate) for estimating the signal propagation delay between channels. Increasing the sampling rate may improve the accuracy of MFCV estimation.

The correlation between torque and EMG spectrum was examined in this study. We found no significant correlation between the averaged MF or MPF and torque for both paretic and contralateral muscles. In an earlier study of the FDI muscle in stroke subjects, we found no correlation between averaged MPF and contraction force (10–80% MVC) in both paretic and contralateral muscles. The recruitment range of biceps brachii motor units is wide with motor units being recruited up to 80% MVC. By contrast, the recruitment range of FDI motor units is relatively narrow (up to approximately 40% MVC), so that production of large forces primarily relies on rate modulation. Given the recruitment pattern difference between the FDI and biceps brachii muscles, we expected a more clear correlation between muscle force and EMG spectrum to be observed in this study. However, our results indicate no significant correlation between the averaged MF or MPF and torque for both paretic and contralateral muscles. This result implies that the relation between muscle contraction level and EMG power spectrum is not necessarily related to small or large muscles. After stroke, complex neuromuscular changes might further compromise the relation between muscle force and EMG spectrum. For example, the study by Kallenberg and Hermens (2009) found that the MF of the contralateral biceps brachii muscle increased slightly between 5 and 50% MVC forces, whereas the MF decreased on the paretic side across the same force range.

The results of the present study also showed that the surface EMG MF and MPF over the estimated innervation zone was significantly larger than more proximal or distal locations for the tested stroke subjects. This is consistent with previous studies (Farina et al., 2001; Beck et al., 2008). These findings confirm that the EMG power spectrum can be used to estimate the location of innervation zone. An interesting finding of this study is that the linear electrode array was placed based on anatomical symmetry, which did not result in similar identified innervation zone locations for paretic and contralateral muscles of the same stroke subjects. This finding demonstrates the importance of using a linear electrode array for EMG examination. Taking advantage of an electrode array, the muscle innervation zones can be estimated and considered for data analysis and interpretation. Otherwise, the uncertainty of the surface electrode location with regard to innervation zone will compromise the interpretation of physiological factors. In addition, a linear electrode can record many channels over a large portion of a muscle, thus providing a more comprehensive examination of the muscle compared with single channel surface electrodes.

In summary, the relationships of the EMG power frequency, MFCV, torque, and electrode location for both paretic and contralateral muscles of stroke subjects were investigated in the present study. The findings indicate complex central and peripheral neuromuscular alterations which can collectively reduce the MF, MPF, or MFCV of surface EMG. No significant correlation between the averaged MF, MPF, or MFCV and torque was found for both paretic and contralateral muscles. The electrode position with respect to innervation zone location appeared to be important for the EMG power spectral analysis. The findings from the linear electrode array examination of stroke subjects provide novel insights and can supplement single channel-based surface EMG analysis for better understanding of complex neuromuscular changes post-stroke and relevance of electrode position for signal analysis and interpretation.

## Conflict of Interest Statement

The authors declare that the research was conducted in the absence of any commercial or financial relationships that could be construed as a potential conflict of interest.
